# Synthesis of a Bioactive Nitric Oxide-Releasing Polymer from S-Nitrosated Starch

**DOI:** 10.1021/acsomega.4c03255

**Published:** 2024-09-23

**Authors:** Jéssica Fernanda da Silva, Edson Araujo de Almeida, Geovana Ellen Karoleski, Everton Koloshe, Ana Paula Peron, Aldo Eloizo Job, Fernanda Vitória Leimann, Marianne Ayumi Shirai, Regiane da Silva Gonzalez

**Affiliations:** † Food Engineering Course, Federal Technological University of Paraná (UTFPR), Campo Mourão Campus, Campo Mourão 87301-899, Paraná, Brazil; ‡ Post-graduation Program of Chemistry, State University of Maringá (UEM), Maringá 87020-900, Paraná, Brazil; § Chemical Engineering Course, Federal Technological University of Paraná, Campo Mourão 87301-899, Paraná, Brazil; ∥ Chemical Course, Federal Technological University of Paraná, Campo Mourão 87301-899, Paraná, Brazil; ⊥ Department of Biodiversity and Nature Conservation, Federal Technological University of Paraná, Campo Mourão 87301-899, Paraná, Brazil; # Department of Physics, State University Paulista “Julio de Mesquita Filho”, Campus, Presidente Prudente 19060-900, São Paulo, Brazil; ∇ Postgraduate Program in Food Technology, Federal Technological University of Paraná, Campo Mourão 87301-899, Paraná, Brazil; ○ Department of Chemistry, Federal Technological University of Paraná, Campo Mourão 87301-899, Paraná, Brazil

## Abstract

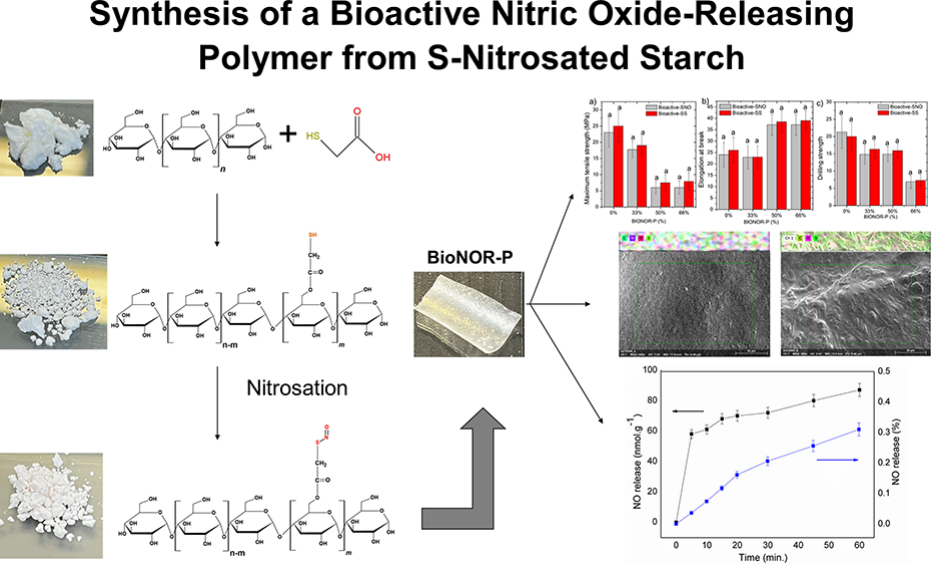

The incorporation of nitric oxide (NO) into polymeric matrices minimizes degradation and facilitates controlled release. This optimization increases the field of application of NO, in dressings, food protective films, and implant devices, among others. This work presents an economical and easy way to manufacture bioactive nitric oxide-releasing polymer (BioNOR-P) and evaluates its bactericidal and antioxidant activity (AA), mechanical behavior, cytotoxicity, and genotoxicity, seeking future use in different applications. The BioNOR-P film was obtained by a casting method, forming a homogeneous, transparent film with good mechanical properties. The release of NO in an aqueous medium showed the film’s ability to release NO slowly, at a rate of 0.58 nmol/g^–1^ min^–1^. Furthermore, the noncytotoxicity and antioxidant activity observed by NO release from BioNOR-P, as well as the ability to inhibit bacterial growth, may aid in the development of a NO-released polymer with different areas of application.

## 1 Introduction

Currently, many studies have been conducted for the development of bioactive materials for several applications in many fields, such as in the pharmaceutical industry, with dressings for skin wounds, as well as other biomedical and healthcare products; in food science, as packaging; and in building materials science, as paint, for examples.^1−3^ Several types of bioactive polymers have been developed due to their characteristics as an adjuvant in the healing process, reduction of oxidative action, and their antimicrobial properties applied to wound healing. Furthermore, they also apply to biodegradable and/or antimicrobial packaging, bioactive coatings and paints, microbe-repellent, and antireflective surfaces.^3−6^ Regarding antimicrobial activity, bioactive materials have some advantages over conventional products, as they can contribute to reduce microbial proliferation and contamination, in order to reduce cost and to increase human health quality.^1,7^

The physicochemical, toxicological, and diffusion properties of these materials depend on their constitution. In this sense, it is possible to highlight the materials known as hydrogels, semipermeable films, foams, hydroactives, biofilms, oil-derived polymers, hydrofibers, hydrocolloids, and nanomaterials.^4−6,8^ Some studies have been carried out using polymers containing nitric oxide (NO) donors, which potentiates the bioactive effect of polymer material.^7,9−11^ These materials have antifouling, antithrombotic, and antimicrobial properties, and can be used in medical and nonmedical materials, such as food packaging applications.^7,12−14^

Nitric oxide (NO) is a radical molecule with a short half-life and rapid inactivation by reactive species contained in plasma and the cellular environment such as superoxide radicals. However, when incorporated into carrier molecules, NO has its biological properties and functions preserved and greater stability, making its application viable. NO is responsible for triggering many of the chemical-physiological and pathophysiological processes,^9,15,16^ has vasodilating properties capable of regulating blood pressure, in addition to regulating several other important processes in mammals, such as macrophage-mediated cytotoxicity, platelet adhesion, cell proliferation and differentiation, and, if present in high doses, it also has germicidal and bactericidal action, which may be highly cytotoxic and can be used to treat tumors.^16−19^

Despite several classes of compounds (e.g., *S*-nitrosothiols,^9,18^*N*-diazeniumdiolates,^9^*N*-Nitrosamines,^20,21^ etc.) that have the potential to transport, release NO, and decompose, we highlight the *S*-nitrosothiols (RSNO) as NO donors.^16,18−20^ In general, *S*-nitrosothiols are easily formed, have potential roles in biological signaling, and can act as trans-nitrosating agents (transfer of a NO^+^ group).^16^ Moreover, thiol groups can be incorporated into polymeric matrices forming bioactive polymers based on polysaccharides (e.g., chitosan and dextran).^18,22^ In addition, polysaccharide-based materials can be used to engineer many physical materials such as hydrogels, films, fibers, coatings, membranes, etc.^8,10,11,13,18,23,24^

Since the ability of the bioactive polymer confers an important role for specific function, it is necessary to highlight that the therapeutic effect of NO release can be evaluated through several physiological mechanisms,^21,24−26^ which are dose-dependent.^26,27^ These researches have been focused on developing NO-releasing devices not only in order to control the amount of NO released at target sites while limiting cytotoxicity but also to improve NO bioactivity and kinetics.^12^

However, the vast majority of these materials are encapsulating matrices of NO donor molecules.^17,18,24,26^ On the other hand, there is little research that uses modified polymers as NO releasers, with emphasis on research targeting antibacterial applications and pharmacological activity.^18,22^ In this present research, the effect of NO release from a bioactive nitric oxide-releasing polymer (BioNOR-P) was evaluated at the cellular level, in order to determine its antioxidative action and cellular toxicity, and at the microbiological level, to determine its microbicidal action. Additionally, we examined the mechanical behavior of BioNOR-P in film forms. Thus, the bioactive material produced can be applied as bioactive dressing, packaging, and edible coatings, among others.

In this sense, this research’s differential is the development of bioactive materials obtained from starch (S), a low-cost renewable raw material. Furthermore, its production uses simple synthesis routes with low production costs, generating a product with high mechanical properties, microbicidal activity, and capacity to reduce oxidative stress. The obtained product has a high added value for use as a bioactive polymer, in the form of bioactive films for use in wound dressings, bioactive packaging, or microbicidal coatings.

## 2 Materials and Methods

### 2.1 Bioactive Polymer Synthesis and Characterization

**Figure 1:**
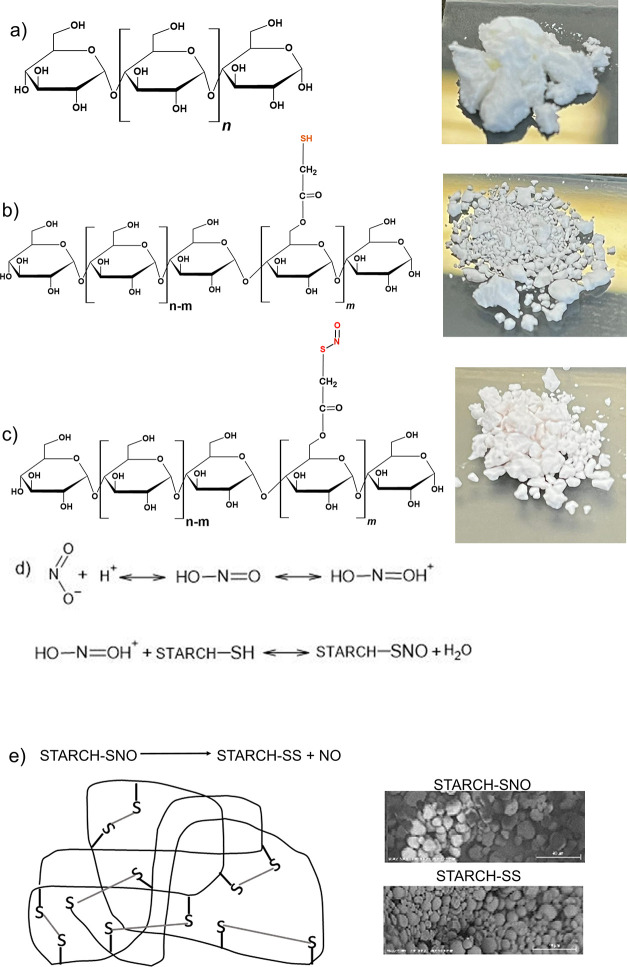
Schematic representation, photographs, and scanning electron microscope (SEM) (magnification/40 μm) analysis of (a) starch (S), (b) thiolated starch (TS), and (c) S-nitrosated starch forming the BioNOR-P. Thiol groups present in TS are colored in dark yellow, and S–NO bound in BioNOR-P is colored in red. (d) S-nitrosation reaction representation. (e) Disulfide linkage between the starch thiomers as a result of the NO release. Photograph courtesy of Emanuel Vinicius Crisostomo de Souza.

The bioactive NO-releasing polymer (BioNOR-P) was prepared at the chemistry laboratory of the Federal University of Technology, Parana State, Campus Campo Mourão–PR. Initially, the chemical modification of starch (Figure 1a) was performed through the grafting process of thioglycolic acid molecules by esterification reaction.^28^ For this, a homogeneous solution of starch (S) was prepared in dimethyl sulfoxide (DMSO) (10% w/V). For each 100 mL of solution, 400 μL of HCl (PA) and 600 μL of thioglycolic acid were added. The reaction mixture was kept under stirring and heated at 60 °C for 2 h. Subsequently, the solution was cooled to room temperature, and thiolate starch (TS) (Figure 1b) was separated from the solvent by precipitation in acetone. The precipitate was washed three times in acetone for DMSO removal, and then dried and stored under 10 °C refrigeration. The grafting process by esterification was evaluated by infrared spectrophotometry.

However, the S-nitrosated starch (BioNOR-P) (Figure 1c) was obtained by preparing a thiolate starch solution (TS) 5% in an acidic medium (pH = 1) in the presence of sodium nitrite (75g/L in HCl 1 mol/L).^30^ After 5 min, the solution was neutralized with NaOH. Through this process, the thiol groups were nitrosated and formed S-NO groups.

The microstructure of powder starch was revealed by a Hitachi SU3800 microscope. A small amount of powder was prepared on stubs covered with double-sided carbon tape and metalized with a thin gold layer. Powder samples were examined by using an accelerating voltage of 9 kV.

#### 2.1.1 NO Evaluation Profile and Antioxidant Activity (AA) Determination

The concentration of NO present on pure BioNOR-P was evaluated in aqueous solution and at room temperature, using Griess reagent.^29^ For this, a known mass of the material was immersed in an aqueous solution (phosphate-buffered saline (PBS), pH 7.4) whose concentration of NO was evaluated spectrophotometrically at 540 nm at 0, 5, 10, 15, 20, 30, 45, and 60 min. After this period, through the use of a HgCl_2_ solution in DSMO, the total content of NO released per gram of BioNOR-P was determined.

Total antioxidant activity was determined at room temperature by the free radical capture method, 1,1-difenil-2-picrilhidrazil (DPPH)^30^ for the BioNOR-P, in concentrations of 2, 0.2, 0.02, and 0.002%, m/V. For this, 0.1 mL of each BioNOR-P solution was added to 3 mL of a DPPH solution at a concentration of 0.00316% (m/V). The mixture was left to rest in the dark for 30 min and then evaluated spectrophotometrically at 515 nm. The ability to eliminate the DPPH radical was used to determine the percentage of antioxidant activity (AA %), which was calculated using the following equation
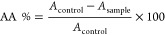
1where *A*_control_ is the absorbance of the DPPH solution without the sample and *A*_sample_ is the absorbance of the sample with DPPH.

### 2.2 *Allium cepa* Test System Considerations

The evaluation of cytotoxicity and genotoxicity in *A. cepa* is used worldwide for the evaluation of chemical compounds/substances, such as natural and synthetic medicines, food additives, cosmetics, and contaminants.^31−34^ Its wide use is due to the intense cell proliferation that occurs in the meristematic region of its roots, which allows the evaluation of the cell cycle, mitotic spindle alterations, and chromosomal breaks.^35,36^ The results obtained through this bioassay can be extrapolated to other eukaryotic organisms since the cellular control of interphase and mitosis are very similar between them.^37^ In addition to the kinetics of cell proliferation, its wide use is also due to its reduced number of chromosomes (2*n* = 16), which facilitates cytogenetic analysis.^36^ The results obtained by means of the bioassay with *A. cepa* have a good correlation with the results observed in genetic tests carried out in other bioassays, such as those with mammals and in cell culture.^38,39^

#### 2.2.1 Obtention, Rooting of Bulbs, and Test of Cytotoxicity and Genotoxicity in *A. cepa* Roots

For the BioNOR-P cytotoxicity and genotoxicity assessment, low concentrations of NO were defined (0.5; 1.0; 2.5, and 5.0 μmol L^–1^), which are known to present antioxidant and anti-inflammatory activities.^40,41^ To determine the cytotoxicity and genotoxicity^42^ in *A. cepa*, the solutions were prepared by BioNOR-P dissolution in deionized aqueous.

For the assessment of the BioNOR-P solutions, onion bulbs (variety Baia Periforme, obtained from an organic garden) were placed in bottles with deionized water and constantly aerated until roots were 2.0 cm in length. For the analysis of each treatment (control groups and BioNOR-P concentrations), an experimental group with five onion bulbs was established.

#### 2.2.2 Cytotoxicity and Genotoxicity Assay

After obtaining roots of 2.0 cm in length, some roots were collected and fixed to serve as control of the bulb itself, which was identified as time of analysis 0 h or control of the bulb itself. Then, the remaining roots were placed in contact with their respective treatment solutions for 24 and 48 h (procedures called exposure times 24 and 48 h), where root collection was performed every 24 h.

A positive control (Co^+^) was prepared with methylmethanesulfonate (MMS), a substance known to be genotoxic to the *A. cepa* test system at 4 × 10^–4^ mol/L. Five bulbs previously rooted in distilled water kept in contact with the MMS solution for 24 h, and then these roots were collected. All roots collected during the cytotoxicity and genotoxicity experiment were fixed in Carnoy 3:1 (ethanol: acetic acid) for up to 24 h.

Glass slides were prepared and analyzed under an optical microscope with a 40× objective lens.^33^ For each bulb, 1,000 cells were analyzed, totaling 5,000 cells for each control group (0 h), with each group’s exposure time of 24 h, and totaling 15,000 cells analyzed for each concentration of BioNOR-P, with each group’s exposure time of 48 h. Similarly, 5,000 cells were analyzed for the MMS group. Cells in interphase, prophase, metaphase, anaphase, and telophase were counted, and the mitotic index (MI) was calculated according to eq 2
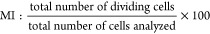
2

Genotoxicity was defined based on the cell change index (CCI), which is characterized in 500 cells analyzed for each analysis time (eq 3) adding 1,500 cells for each treatment. The categories of aberrant cells considered were micronucleus, stickiness, and abnormal ana/telophases, which include bridges, vagrant chromosomes, chromosome missegregation, and multipolar spindles. The presence of cells undergoing apoptosis was also observed.
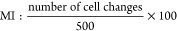
3The cytotoxicity and genotoxicity data obtained were tested by the Kruskal–Wallis test (*H* test), followed by the Dunn’s test (*p* ≤ 0.05), using the software Rstudio 1.3.

### 2.3 Preparation, Microbicide, and Microscopic and Mechanical Characterization of BioNOR-P Film

Initially, the thiolated starch (TS) film was prepared by casting process^43^ using 3 g of a mix of starch (thiolated starch + acetylated starch) and glycerol as plasticizer (0.6 g). Four formulations with different TS contents (0, 33, 50, and 66%) in relation to the total starch mass were studied. The mixture of starch and glycerol was solubilized in water (96.4 mL) at 75 °C under manual stirring in a water bath (WEA-model 837-2). Then, the product was spread in a Teflon-coated mold (25 cm × 37 cm) and dried in an oven with air circulation (Novaética, Vargem Grande Paulista–SP, Brazil) at 40 °C/22 h.^44^ After drying, the films were nitrosated,^24^ by being cut into pieces of known mass and area and then immersed for 3 min in an acid solution (HCl, 1 mol/L) of sodium nitrite (0.14 mol/L), thus giving rise to the BioNOR-P film. After nitrosation, the films were washed three times with deionized water in order to remove reagent excess. The NO release profile was evaluated in aqueous solution (PBS, phosphate-buffered saline, pH 7.4) and at 25 and 37 °C, as described in Section 2.1.1.^34^ The microstructure of films was revealed by SEM/energy-dispersive X-ray spectroscopy (EDS) (Hitachi SU3800 microscope/Bruker XFlash EDS) in accordance with Section 2.1. Acceleration voltages varying through 5–7 kV were used for the cast film.

To evaluate the stability/solubility of BioNOR-P films in buffer solution (pH 7.4), a piece of (5 mm × 5 mm) was transferred to buffer solutions at 37 °C, and at regular intervals (0.5, 1, and 24 h), its material was collected and its weight was measured.^45^ The stability/solubility of BioNOR-P films was expressed as percent of the lost mass of starch (% solubility) relative to the initial solid weight of the film.

#### 2.3.1 Evaluation of the Microbicidal Activity of BioNOR-P Films

BioNOR-P films were evaluated for antibacterial potential by the disk diffusion method.^44^ The bacteria *Staphylococcus aureus* and *Escherichia coli* were used, both obtained from the Food Microbiology Laboratory, Federal Technological University of Paraná, UTFPR, Campo Mourão Campus. The bacteria were seeded in Petri dishes on BPA (Baird Parker Agar) medium for *S. aureus* and EMB (Eosin Methylene Blue) agar for *E. coli*. On each quadrant of the plates sown with bacteria, disks of acetylated and nitrosated starch films were applied, both with a diameter of 7 mm. The experiment was performed in triplicate for each bacterial culture. The plates were incubated at 37 °C for 48 h in a microbiological incubator. After the incubation period, the size of the zone of inhibition (ZOI) was measured using a caliper.^44^

#### 2.3.2 Assessment of the Mechanical Resistance of BioNOR-P Films

Assessment of the mechanical resistance of BioNOR-P films was realized to bioactive films after nitrosation reaction and to films after total NO release induced by light exposition until no more NO detection by the Griess assay was observed. The BioNOR-P films were cut into dimensions of 50 mm × 20 mm and conditioned in desiccators containing saturated magnesium nitrate saline solution (53% equilibrium relative humidity) at 25 °C for a period of 48 h.^46^ After conditioning, the tensile tests were carried out using a texturometer, Stable Micro Systems, model TA XTplus (England), with an initial claw distance of 50 mm and a tensile speed of 0.8 mm s^–1^. The determined properties were maximum tensile strength (MPa) and elongation at break (%). A total of five measurements were taken for each formulation.

The maximum tensile strength (*T*) is the relationship between the maximum force (*F*_max_) measured and the initial area (*A*) of the specimen, which is calculated with the width (*L*) and thickness (*e*) values of the test specimen by using eq 4.
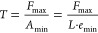
4

The elongation at break (*E*) is the percentage relationship between the elongation (*E*_rup_) of the specimen at break and its initial length, which corresponds to the distance between the claws (*D*_claws_), as shown in eq 5.
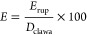
5

The drilling test^41^ was used to determine the force (*N*), in which 20 mm × 20 mm specimens were fixed to a circular support of the texturometer (Stable Micro Systems, model TA XTplus, England), and a metal probe with a spherical tip with a diameter of 6.35 mm at a speed of 25 mm/min drilled the samples.^47^

## 3 Results and Discussion

### 3.1 Development of Bioactive Polymer

This research used a simple method of modification by grafting starch, a low-cost polymer, making it possible to covalently link nitrosothiols to the starch structure, giving rise to a NO-releasing matrix. The use of NO release systems through the incorporation of low-molecular-weight molecules, such as *S*-nitrosothiols,^18,48^ in polymeric matrices has been common due to the ease of reaction between thiol groups (RSH) and NO derivatives, such as NO_2_, N_2_O_3_, among others.^49^

**Figure 2:**
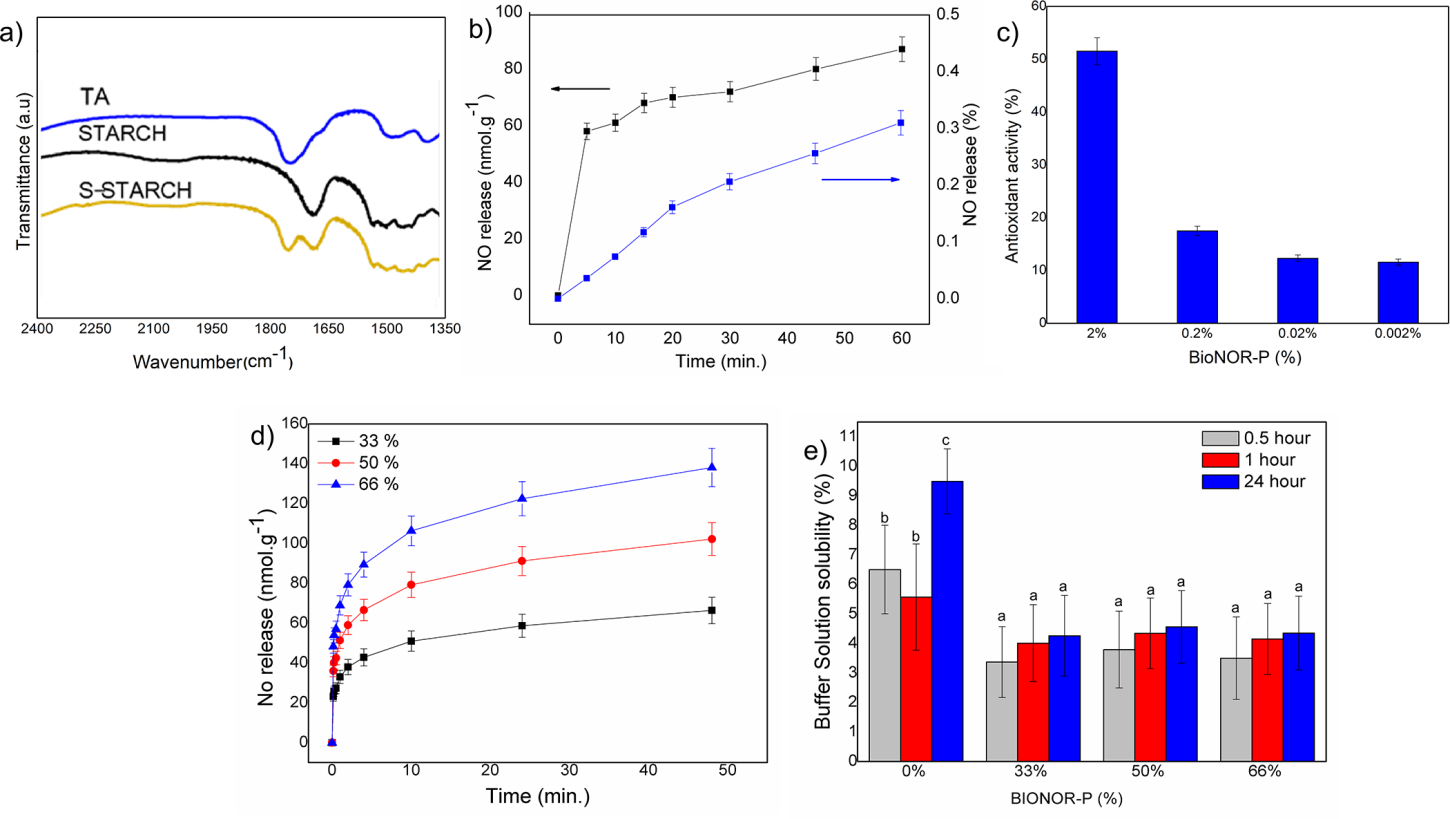
(a) Infrared spectrum for thioglycolic acid (TA), pure starch (Starch), and thiolated starch (S-Starch). (b) NO release profile from bioactive nitric oxide-releasing polymer (BioNOR-P) at 25 °C in the PBS solution (black-left *y*-axis) and percentage of the total NO molecule available in the sample and released during 60 min (blue-right *y*-axis). (c) Antioxidant activity (AA %) of NO released from BioNOR-P in aqueous solution in concentrations of 2, 0.2, 0.02, and 0.002%, m/V. (d) NO release profile from BioNOR-P films in PBS solution at 37 °C in accordance with TS content. (e) Stability/solubility of BioNOR-P films in buffer solution at 25 °C. Equal superscript letters mean equality between the solubility of the films at different times of immersion in buffer solution, after analysis by Kruskal–Wallis (*H* test) followed by Dunn’s test *p* < 0.05.

For this synthesis process, the starch donor of NO (BioNOR-P) was obtained first of all by chemical modification by grafting (Figure 1) with thioglycolic acid followed by nitrosation. In this process, thioglycolic acid molecules are covalently linked to the starch chain through the esterification reaction, forming the thiolate starch (TS). Grafting starch with thioglycolic acid was proven by infrared spectroscopy (Figure 2a). As can be clearly observed in Figure 2a, the peak at 1720 cm^–1^ corresponds to the carbonyl of esters (S-Starch) derived from thioglycolic acid.^50,51^ In (TA), the signal for carbonyl of pure thioglycolic acid is observed at 1700 cm^–1^.^52^ The peak at 1650 cm^–1^ corresponds to the stretching of the −OH bond present in the starch molecule without modification.^53^

After the grafted starch acquires SH groups, there is the possibility of S-nitrosation, and this reaction can be expressed according to Figure 1d. In this research, the thiol groups from TS were nitrosated, forming STARCH-SNO, in aqueous medium through the reaction with nitrous acid formed from the nitrite mixture in an acidic medium, in which STARCH represents the constituent molecules of the modified starch containing the SH group. After STARCH-SNO releases NO, there is the formation of a disulfide linkage between the Starch thiomers (Figure 1e). This bioactive NO-releasing polymer (BioNOR-P) was developed to obtain a matrix derived from a cheap and simple process aiming to release NO directly on the application site.

### 3.2 NO Evaluation Profile and Antioxidant Activity Determination

The quantification of the formed nitrosothiol groups and kinetic profile were evaluated spectrophotometrically at 542 nm using Griess reagent (Figure 2b). Moreover, it was verified by total NO decomposition through HgCl_2_ that each gram of pure BioNOR-P was able to release 160 μmol of NO. Furthermore, in 1 h of analysis, it was observed that 1 g of BioNOR-P films at 33, 50, and 66% TS contents were able to, respectively, release about 29, 43, and 58 nmol of NO (Figure 2d). Moreover, in the literature, there are studies describing different amounts of NO release through different polymeric matrices,^50,54−56^ demonstrating that the development of materials that act as a matrix for NO donation through polymeric materials can become an alternative for the creation of new bioactive materials, which can be applied as wound dressings,^56^ coatings^57,58^ (paints), implant devices,^59^ bioactive packaging,^14^ seed germination and growth,^60^ edible food packing,^7^ among other applications.

From this point, the insertion of nitrosated starch into the matrix led to the formation of a bioactive nitric oxide-releasing polymer, whose NO release profile in the first hour of release in aqueous medium can be observed in Figure 2b. As can be seen from the figure, regardless of the initial burst caused by the immersion of the matrix in water, the NO release rate is slow since it corresponds to 0.58 ± 0.03 nmol·g^–1^·min^–1^. This result indicates that, in the case of in vivo application of the dressing, the release of NO is guaranteed for a long period, which favors biological processes, such as antioxidative processes. Through the DPPH assay, at a 95% level of significance, the antioxidant and dose-dependent action of NO released from BioNOR-P was verified, which showed the capacity to eliminate the DPPH radical between 50 and 12% for the concentrations used (Figure 2c). Considering that this bioactive polymer was projected to release small amounts of NO (Figure 2b), it is possible to infer that at experimental conditions, an increase of 0.32 pmol to 0.32 nmol of NO released in solution was able to improve the AA % from 12 to 50%.

In animal or plant cells, the redox-regulated processes are extremely relevant, and it can be reached through treatment with antioxidants, such as nitric oxide (NO), that is able, in animal cells, to scavenge superoxide, which is the main component of oxidative stress, and in plant cells, it is involved in the cellular redox balance under senescence and hypoxia conditions.^7,61^ The NO, for example, act on angiogenesis at the proliferative phase, on wound healing,^61−64^ and on seed germination during water deficit, promotes greater activity of antioxidative apparatus enzymes and amylases, reducing the damage caused by redox stress,^57^ even as, on edible food packaging, prolonging horticultural products shelf life during storage or different types of environmental stress.^7^

### 3.3 Nitric Oxide Effect on Cellular Levels: Cytotoxic and Genotoxic Analysis of *A. cepa*

**Figure 3:**
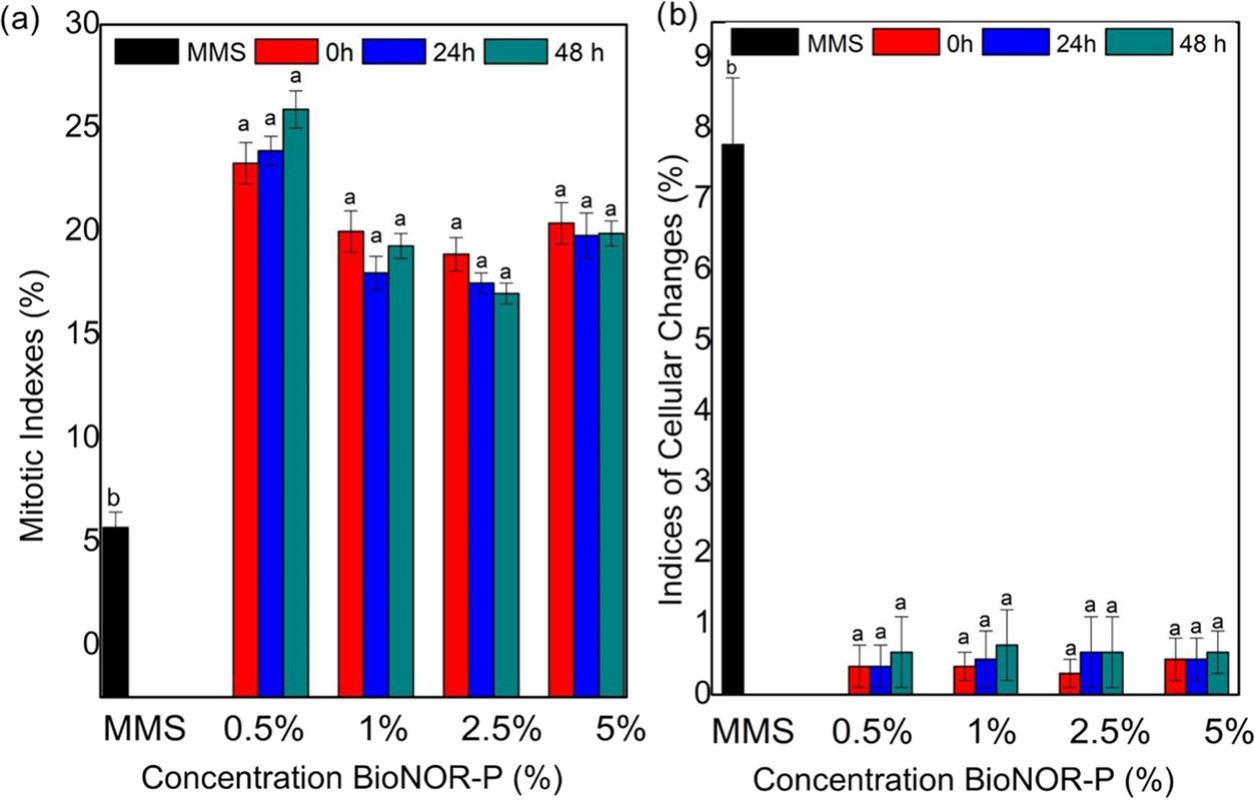
(a) Mitotic indexes and (b) indexes of cellular changes obtained in meristems of *A. cepa* roots exposed to treatments with 0.5; 1.0; 2.5, and 5.0 BioNOR-P in 24 and 48 h of exposure at room temperature; h: hour, MMS: methylmethanesulfonate (positive control). Equal superscript letters signify equality between the exposure times of each concentration after analysis by Kruskal–Wallis (*H* test) followed by Dunn’s test *p* < 0.05.

Regarding cytotoxic and genotoxic analysis of *A. cepa*, it was observed that the mitotic indexes for the 0.5; 1.0; 2.5, and 5.0% BioNOR-P in the 24 and 48 h of exposure were equal to the cell division indexes observed for their respective controls. Under the conditions of the established analyses, such results characterize the noncytotoxic effect (Figure 3) of BioNOR-P. In addition, the treatments in the 24 and 48 h exposure did not induce a significant number of changes in the mitotic spindle and chromosomal breaks when compared to their respective controls, showing also to be nongenotoxic. No apoptotic cells were observed.

These results might suggest that NO responses are level-dependent. To make a broad generalization, we conclude that low relative concentrations of NO tend to favor pro-growth and antiapoptotic responses, whereas higher levels of NO favor pathways that induce cell cycle arrest, senescence, or apoptosis.^24^

In this article, the effect of NO at the cellular level was evaluated, since the response of the body to the application of NO is dependent on both how NO is administered and, on the dose, used. The results reported specific mechanisms of action of NO, and also they highlight the dichotomous nature of NO under various biological conditions. In very small doses (<1–30 nM), NO acts as a signal of cGMP, and in this case, it can mediate proliferative and protective effects.^19,64^

These levels are lower in endothelial cells in which NO fluxes less than 1 nM can induce proliferation. As the NO levels increase, HIF-1α is stabilized, so NO concentrations can have a proliferative response and can confer protection against tissue injury. However, at higher concentrations, it does not induce phosphorylation, which may have a cytostatic or even an apoptotic response.

### 3.4 Antibacterial Potential of BioNOR-P Films

Another very important property of NO is its broad antibacterial action, acting against Gram-positive and Gram-negative strains.^49^ It is reported in the literature that low concentrations of NO are capable of stimulating the immunological activities of cells,^19^ while high concentrations induce death of bacteria by different mechanisms, such as nitrosative and oxidative stress of proteins and lipids, membrane rupture, among others.^64^

**Figure 4:**
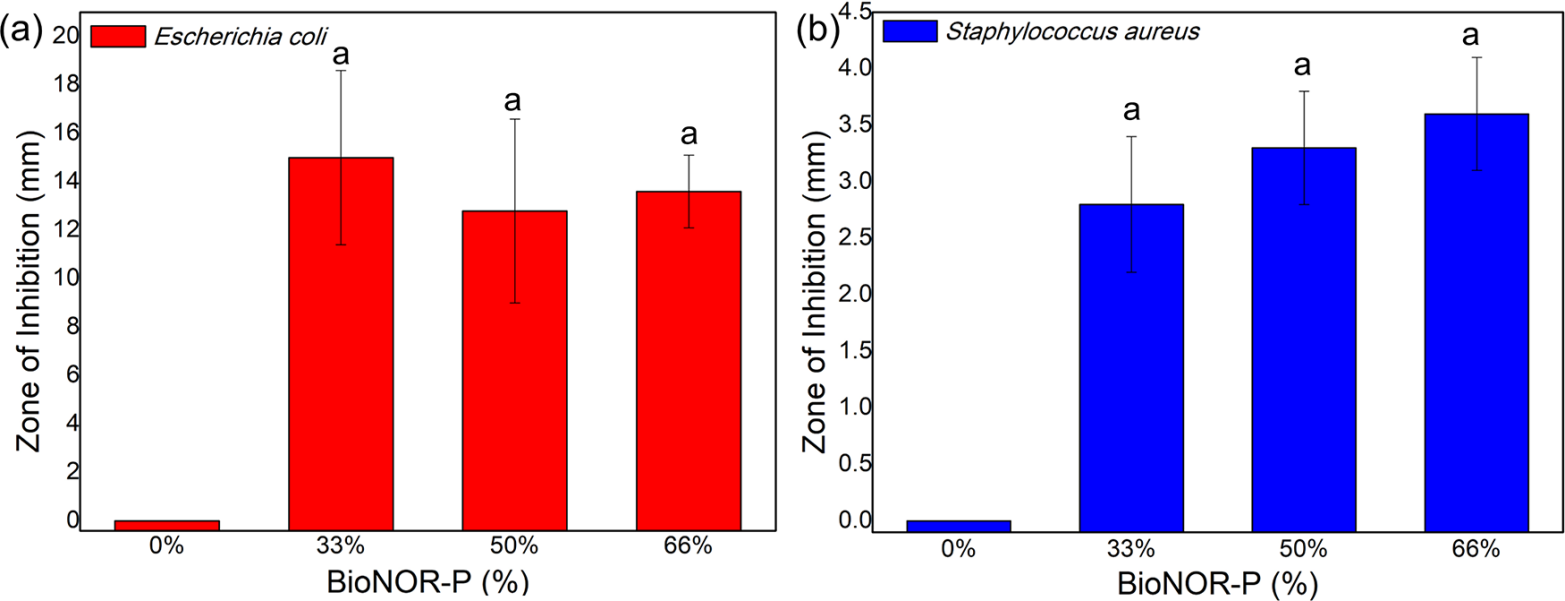
Zone of inhibition (ZOI) graph of bioactive nitric oxide-releasing polymer (BioNOR-P) films against (a) *E. coli* and (b) *S. aureus*, through plate incubation at 37 °C for 48 h. Equal superscript letters signify equality between the antibacterial potential of each BioNOR-P (%) film after analysis by Kruskal–Wallis (*H* test) followed by Dunn’s test *p* < 0.05.

In this research, it was verified that all BioNOR-P films showed inhibitory action against *S. aureus* and *E. coli*. Figure 4 shows the measurements of the ZOI of the BioNOR-P films, which ranged from 3.0 to 3.6 and from 9.6 to 15 mm, respectively, for *S. aureus* and *E. coli*. However, no increase in the bactericidal activity was observed for the film containing the highest content of S-nitrosated starch.

In this research, it was verified that all BioNOR-P films showed inhibitory action against *S. aureus* and *E. coli*. A similar study shows the bactericidal activity of a water-soluble NO-releasing polysaccharide derivative based on carboxymethylated dextran and cysteamine, where NO release results in reduction in viable *E. coli*, *Acinetobacter baumannii*, and *S. aureus* in nutrient broth media.^22^ The ZOI measurements of the BioNOR-P films for *E. coli* and *S. aureus* are shown in Figure 4. The BioNOR-P films presented zone of inhibition measurements ranging from 3.0 to 3.6 and from 9.6 to 15 mm, respectively, for *S. aureus* and *E. coli*. These results indicate that all materials (33, 50, and 66%) present a total NO amount enough to inhibit bacterial growth. However, no increase in bactericidal activity was observed for the film containing the highest content of S-nitrosated starch. In this case, this effect is governed by the NO amount diffused from BioNOR-P film into the agar medium and not by the content of S-nitrosated starch present in BioNOR-P film. Then, it is possible to develop a material with mechanical properties variables as presented in Section 3.6 without changing its microbicidal activity.

Even though the agar diffusion is a method used for determining the susceptibility of bacteria to antimicrobials and considered a nonquantitative analysis,^65^ the lower inhibition in relation to *S. aureus* can be attributed to differences in the structure of the bacterial wall. For example, the presence of lipopolysaccharide in Gram-negative bacteria and its absence in Gram-positive bacteria play a crucial role in determining the bacteria’s permeability to external substances. This structural variation impacts the entry capacity of different compounds, thus influencing the response to inhibition.^66^ As expected, the control formulation did not inhibit the growth of *S. aureus* and *E. coli*, proving that the S-nitrosated S present in the films was mainly responsible for the antibacterial activity. The inhibition capacity of *E. coli* and *S. aureus* bacteria by NO donor polymeric materials is widely discussed in the literature,^49,67,68^ corroborating the results found in this work.

### 3.5 Microstructure of BioNOR-P Films and Stability in Buffer Solution

**Figure 5:**
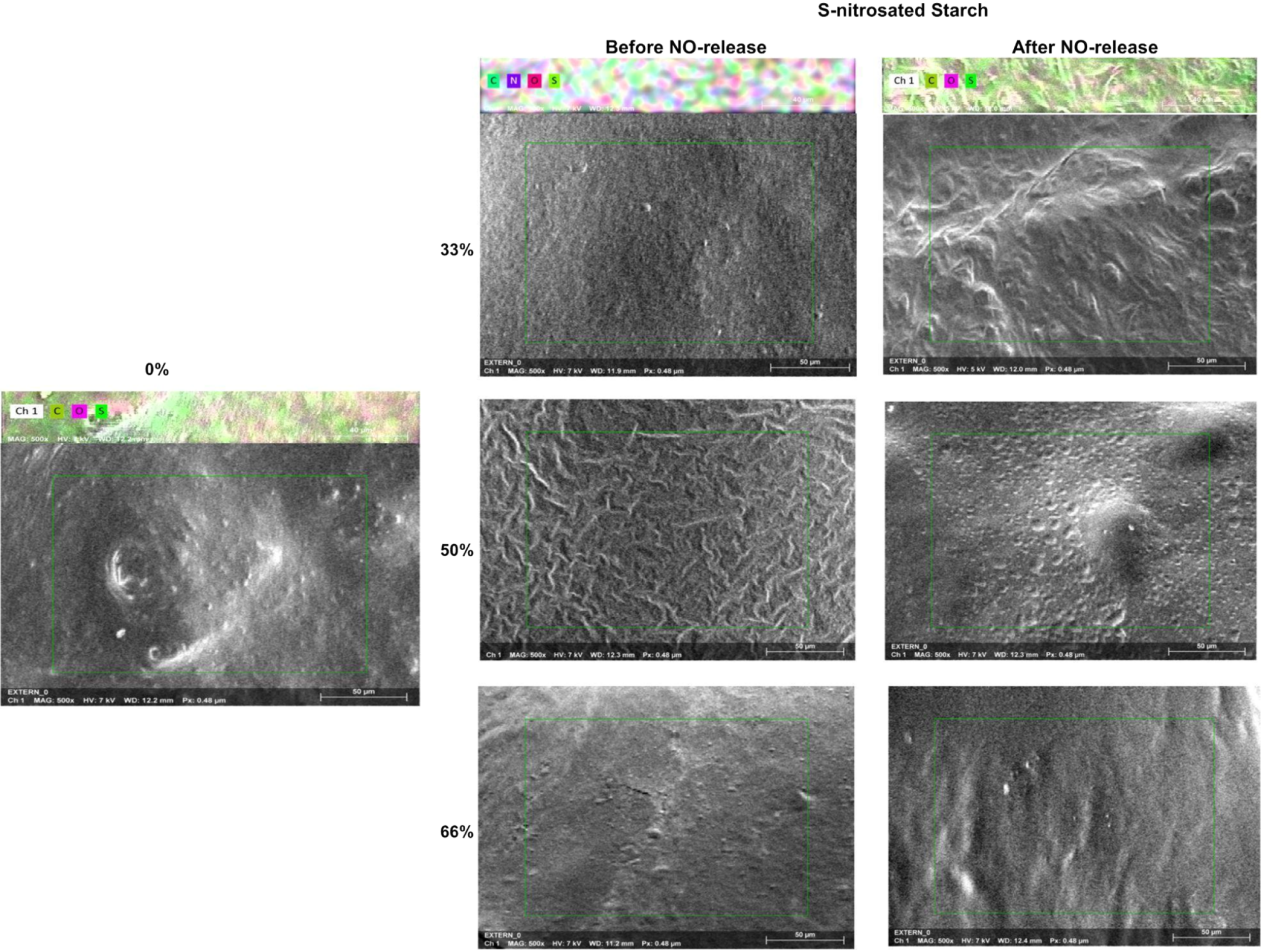
Microstructural observation by SEM/EDS of modified corn starch films in accordance with the BioNOR-P content. Magnification: 50 μm between marks.

All BioNOR-P film formulations were transparent, homogeneous, with good handling properties, and were easily removed from the molds after drying. The average thickness of BioNOR-P films was 0.110 mm, being smaller than that found for acetylated starch films in the literature, which reports values between 0.148 and 0.158 mm.^69^ The influence of the TS content and disulfide links in BioNOR-P films was explored by SEM analysis (Figure 5). It was observed that all films present smooth morphology as a result of predominant starch chains in amorphous phase. During the casting process, starch granules stuck to another resulting in no crystalline regions in the films, generally represented by higher surface roughness.^45^ Moreover, there was no significant difference observed among films once all films presented smooth surfaces without pores or cracks. In this case, the presence of plasticizer presents a higher influence on material properties compared to TS content or disulfide links in BioNOR-P films due to low graft content in starch.

The materials preconditioned at 53% relative humidity kept their mechanical integrity. Furthermore, it was observed that the BioNOR-P presents low solubility in buffer solution corresponding to about 5% in films containing BioNOR-P (33, 50, and 66%) and 10% in films without BioNOR-P (Figure 2e). In this case, the solvent molecules were not favorably absorbed by the starch macromolecular network grafted with nitrosothiols, showing the high stability of the chain’s entanglement. Although there is no ideal value, the solubility will determine if the films can be in contact with high water activity materials or to avoid exudation.^45^

### 3.6 Mechanical Properties of BioNOR-P Films

**Figure 6:**
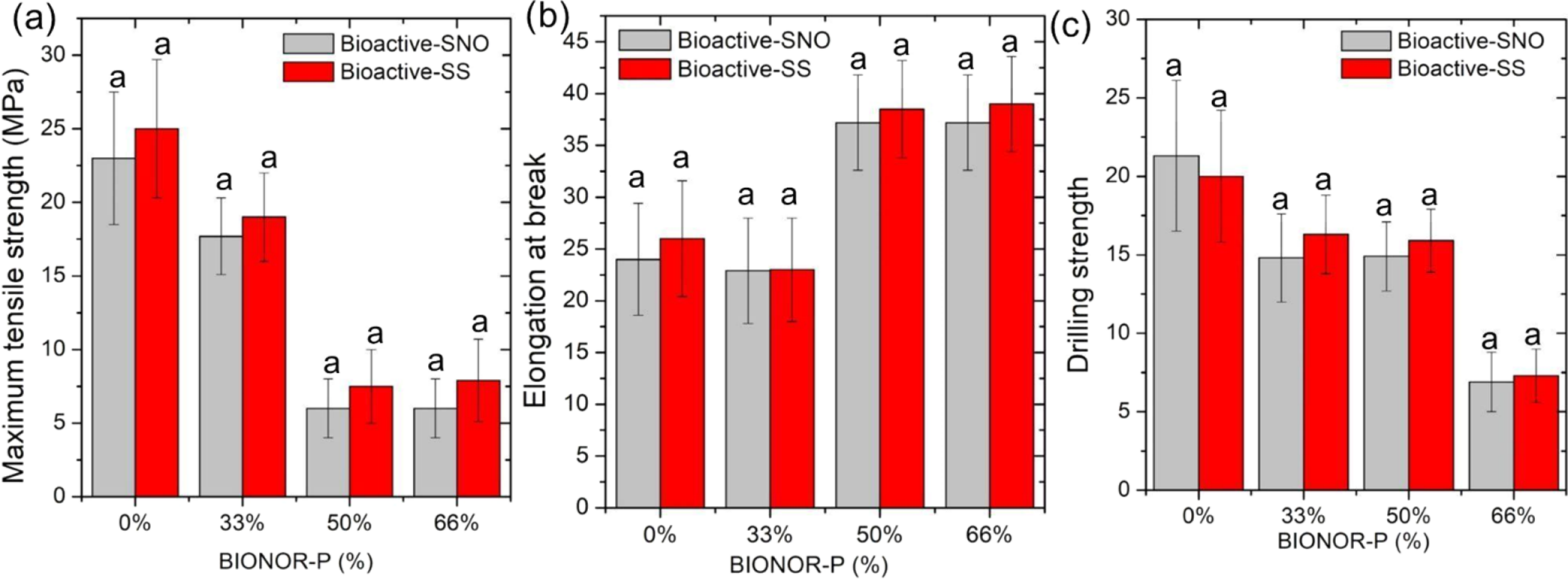
Mechanical properties of modified corn starch films containing S-NO (Bioactive-SNO) and post NO release (Bioactive-SS). (a) Maximum tensile strength, (b) elongation at break, and (c) drilling strength. Equal superscript letters signify equality between the mechanical properties of BioNOR-P (%) film (SNO vs SS) after analysis by Kruskal–Wallis (*H* test) followed by Dunn’s test *p* < 0.05.

The mechanical properties of BioNOR-P films, referring to resistance to traction (*T*), elongation at break (*E*), and force at drilling (*P*), are shown in Figure 6.

For films that contained higher proportions of acetylated starch, an increase in tensile strength and a reduction in elongation at break were observed, that is, high proportions of acetylated starch favor tensile strength and reduce elongation at break.^69^

For the drilling test, lower resistance to perforation was observed with an increase in the concentration of starch modified with thioglycolic acid in its formulation, showing that the acetylation of starch provides films with higher tensile and drilling resistance.

The acetylation process is an esterification reaction that is known to provide starch with a retardation in retrogradation, as well as increase the paste clarity and viscosity.^70^ As a result, films formed with acetylated starch demonstrated a higher strength and resistance to both tension and drilling. In practice, the higher tensile strength in a film typically correlates with lower elongation capacity. However, in this study, it was observed that formulations with a higher content of acetylated starch exhibited greater tensile strength, while formulations with a higher content of S-nitrosated starch showed an increased level of elongation. This indicates that starch modified with thioglycolic acid presents higher plasticity compared with acetylated starch. Moreover, the NO-releasing results in a small increase in starch molecular weight attributed to the formation of disulfide linkage between the starch thiomers (Figure 1e). However, there were no significant changes in mechanical behavior (Figure 6), due to the small amount of S-NO groups present in the material.

Thus, the polymeric NO-releasing film produced with low-cost NO donors for antioxidant and antibacterial applications presents the advantages of targeting ability, stimulating responsiveness, antibiofilm ability, and biocompatibility, as well as adequate mechanical properties to be applied on flexible wearable devices such as bioactive dressing, packaging, and edible coatings.

## 4 Conclusions

In this article, it was possible to develop a bioactive, noncytogenotoxic, antioxidant, and low-cost nitric oxide-releasing polymer, capable of slowly releasing NO (0.58 nmol/g^–1^ min^–1^). Moreover, the evaluated bioactivity demonstrated that BioNOR-P effectively inhibited bacterial growth presenting ZOI ranging from 3.0 to 3.6 and from 9.6 to 15 mm, respectively, against *E. coli* and *S. aureus*. Furthermore, it can be used to compose a polymeric film whose formulations showed good mechanical properties with effective dose dependent on tensile, elongation, and drilling according to TS content. These good mechanical and bioactive properties allow its application for packaging and coating in order to contribute to the reduction of pathological oxidative stress.

These results demonstrate a positive aspect for the future commercial use of BioNOR-P as an exogenous NO donor to be applied on flexible wearable devices such as bioactive dressing, packaging and storage, and maintenance of horticultural products.

## Abbreviations

NO: nitric oxide
BioNOR-P: bioactive nitric oxide-releasing polymer
DMSO: dimethyl sulfoxide
S: starch
TS: thiolate starch
DPPH: 1,1-difenil-2-picrilhidrazil
MMS: methylmethanesulfonate
SEM: scanning electron microscope
EDS: energy-dispersive X-ray spectroscopy
